# Associations of perceived stress with loneliness and depressive symptoms: the mediating role of sleep quality

**DOI:** 10.1186/s12888-024-05609-2

**Published:** 2024-03-01

**Authors:** Xiao Wang, Xingqi Cao, Jiening Yu, Shuyi Jin, Shengyi Li, Liying Chen, Zuyun Liu, Xuan Ge, Yangzhen Lu

**Affiliations:** 1 Department of General Practice, Dongyang People’s Hospital, 322100 Jinhua, Zhejiang China; 2 Center for Clinical Big Data and Analytics Second Affiliated Hospital, Department of Big Data in Health Science School of Public Health, The Key Laboratory of Intelligent Preventive Medicine of Zhejiang Province, Zhejiang University School of Medicine, 310058 Hangzhou, Zhejiang China; 3 School of Mathematical Sciences, University of Nottingham, University Park, NG7 2RD Nottingham, UK; 4 Department of General Practice, Sir Run Run Shaw Hospital, School of Medicine, Zhejiang University, 310016 Hangzhou, Zhejiang China; 5 Health Management Center, Dongyang People’s Hospital, 322100 Jinhua, Zhejiang China

**Keywords:** Perceived stress, Loneliness, Depressive symptoms, Sleep quality, Mediation

## Abstract

**Background:**

Whether perceived stress is associated with loneliness and depressive symptoms in general adults, and to what extent sleep quality mediates the associations, remains unknown. The aim of this study was to estimate the associations of perceived stress with loneliness and depressive symptoms, and the mediating role of sleep quality in these associations.

**Methods:**

Cross-sectional data on 734 participants (aged 18–87 years) were analyzed. Perceived stress was assessed using the 10-item Perceived Stress Scale (PSS-10; range 0–40). Sleep quality was assessed using the Pittsburgh Sleep Quality Index (PSQI; range 0–21). Loneliness was assessed using the three-item short form of the Revised University of California, Los Angeles (UCLA) loneliness scale (range 3–9). Depressive symptoms were assessed using the 10-item Center for Epidemiologic Studies Depression (CESD-10) Scale (range 0–30). General linear regression models, multivariable logistic regression models, and formal mediation analysis were performed.

**Results:**

After adjustment for age and sex, we found that with each 1-point increment in the perceived stress score, both the loneliness score (β = 0.07; 95% confidence interval [CI]: 0.06, 0.08) and depression score (β = 0.45; 95% CI: 0.40, 0.49) increased significantly. Robust results were observed when adjusting for more confounders. Furthermore, sleep quality mediated 5.3% (95% CI: 1.3%, 10.0%; *P* = 0.014) and 9.7% (95% CI: 6.2%, 14.0%; *P* < 0.001) of the associations of perceived stress score with loneliness score and depression score, respectively.

**Conclusions:**

In general Chinese adults, perceived stress was positively associated with loneliness and depressive symptoms, and sleep quality partially mediated these associations. The findings reveal a potential pathway from perceived stress to mental health through sleep behaviors, and highlight the importance of implementing sleep intervention programs for promoting mental health among those who feel highly stressed.

**Supplementary Information:**

The online version contains supplementary material available at 10.1186/s12888-024-05609-2.

## Background

According to the World Health Organization (WHO), approximately 280 million people have depression in 2019 [1]. The global incidence of mental health problems substantially increased by 25% in the first year of Coronavirus disease 2019 (COVID-19) [2]. As an increasingly common mental disorder, depression increases suicide risk and contributes to adverse health outcomes (e.g., dementia [3], cardiovascular disease [4, 5], cancer [6], and mortality [7]). It was estimated that the prevalence of depression among adults in China is 6.8% through the China Mental Health Survey [8]. Similarly, loneliness is also prevalent, especially during the COVID-19 pandemic (2019–2022 years) [9]. Based on the latest report from WHO, about 5-15% of adolescents and 25% of older adults are experiencing social isolation and loneliness [10]. While transient feelings of loneliness are normative [11], chronic loneliness has serious health consequences (e.g., dementia [12], cardiovascular disease [13], and mortality [14]). Studies have shown that young people are lonelier than middle-aged and older adults [15]. Both loneliness and depression are critical public health problems, and finding modifiable risk factors for them is of great significance.

Stress has long been a hot topic in health sciences research, as it influences health not only directly through multiple biological systems (e.g., neuroendocrine and autonomic responses), but also indirectly through changes in health behaviors [16]. Previous studies have linked stress with loneliness [17, 18] and depressive symptoms [19–22]. Although most of them focused on depressive symptoms, research on loneliness is actively underway. Also, the influence of loneliness on mortality is comparable to that of other well-established risk factors (e.g., smoking, obesity, and physical inactivity) [10]. In addition, about half of these studies were conducted in older adults [19, 20], the results may not be generalizable to younger adults. The prevalence of loneliness and depression in younger adults has risen sharply [15, 23], which has been an increasing concern. Adults in younger and middle-aged usually bear high pressure and face huge challenges dealing with work, kids, aged parents, and the constant pull of staying connected, making them susceptible to loneliness and depressive symptoms. Therefore, there is a need to clarify the associations of stress with loneliness and depressive symptoms among younger and middle-aged adults. Moreover, Chinese cultural beliefs (e.g., the power of inner self-control) and Chinese beliefs about traditional medicine (e.g., the integrated body-mind relationship) may exacerbate these mental health problems [24]. Considering the large population in China, such an exploration in general Chinese adults is warranted.

An intriguing query is to identify which factor mediates the effect of perceived stress on loneliness and depressive symptoms. It is highly possible that high stress leads to health behavior changes, which in turn increase the risk of loneliness and depressive symptoms. Perceived stress has been demonstrated to be positively associated with poor sleep quality [25, 26]. High stress may interrupt sleep behaviors through hyperactivation of the hypothalamic-pituitary-adrenal axis and presleep cognitive arousals [25]. Meanwhile, sleep behaviors predict loneliness [27] and depressive symptoms [28]. A few studies have reported that stress influences depressive symptoms through sleep quality among urban older adults, students, or health workers [29–32]. However, to the best of our knowledge, no studies have estimated the extent to which sleep quality mediates the associations of perceived stress with loneliness and depressive symptoms in general Chinese adults.

We conducted this cross-sectional study using baseline data from the ZheJiang longitudinAl Study of Healthy Aging (JASHA), an ongoing longitudinal cohort study of the general adults in Zhejiang province, China. This study aimed to estimate the associations of perceived stress with loneliness and depressive symptoms, and the mediating role of sleep quality in the associations.

## Methods

### Study participants

**Fig. 1:**
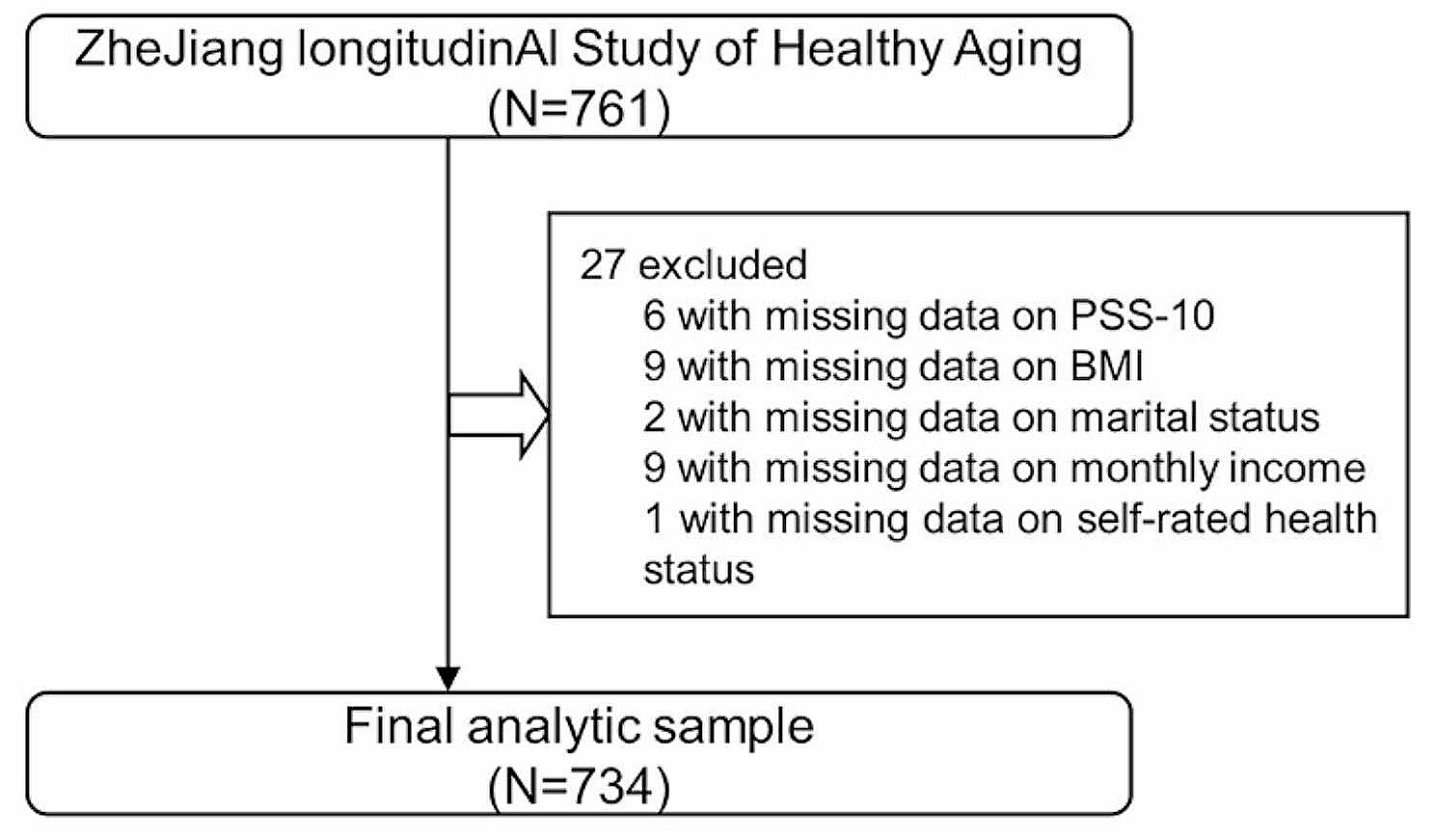
Flow chart of the analytic sample. PSS-10, 10-item Perceived Stress Scale; BMI, body mass index

The JASHA is a prospective, longitudinal cohort study focusing on multi-dimensional aging phenotypes (including physical function, cognitive function, brain health, and mental health) of a general population living in Zhejiang province, China. In this study, we used the samples recruited from Dongyang People’s Hospital’s Health Management Centre (the largest and most comprehensive physical examination center in Dongyang). Dongyang is a city of approximately 1.09 million inhabitants, located in the central region of Zhejiang Province in China. Participants who had a routine annual physical examination at the Dongyang People’s Hospital’s Health Management Centre were invited and recruited. Information including demographics, socioeconomic factors, lifestyle, medical history, and mental health was obtained through face-to-face questionnaire surveys by trained investigators. From June 2022 to June 2023, a total of 761 participants agreed to participate in the study and provided written informed consent. We excluded those with missing data on perceived stress (*N* = 6), body mass index (BMI; *N* = 9), marital status (*N* = 2), family income per month (*N* = 9), and self-rated health status (*N* = 1). The final analytic sample comprised 734 participants aged 18–87 years (Fig. 1). The protocol of this study was approved by the Ethics Committee of the School of Public Health at Zhejiang University and Dongyang People’s Hospital.

### Perceived stress

We measured perceived stress levels using the Perceived Stress Scale (PSS), which was developed by Cohen et al. [33]. PSS has been demonstrated to have good reliability and validity, with Cronbach’s α ranging from 0.78 to 0.91 and test-retest reliability coefficients ranging from 0.55 to 0.85 [33–35]. In this study, we used a simplified Chinese version of PSS — 10-item PSS (PSS-10), which consisted of six negative and four positive items. Each item of PSS-10 was scored on a 5-point Likert-type scale ranging from 0 to 4. The total score of PSS-10 ranged from 0 to 40, with a higher score indicating greater perceived stress levels.

### Sleep quality

We measured global sleep quality over the past month using the Pittsburgh Sleep Quality Index (PSQI) [36]. The PSQI has been widely used to assess sleep quality, with a good internal consistency (Cronbach’s α = 0.83) and test-retest reliability (*r* = 0.85) [37]. PSQI consisted of 19 items that were divided into seven domains: subjective sleep quality, sleep latency, sleep duration, sleep efficiency, sleep disturbance, use of sleep medication, and daytime dysfunction. The score of each domain ranged from 0 to 3. The global PSQI score ranged from 0 to 21, with a higher score indicating poorer global sleep quality. Participants were considered as having poor global sleep quality if they had a PSQI score > 5 as previous studies did [36].

### Loneliness and depressive symptoms

We measured loneliness using the three-item short form of the Revised University of California, Los Angeles (UCLA) loneliness scale [38]. The good internal reliability of the UCLA loneliness scale has been verified (Cronbach’s α = 0.78) [39]. Participants were asked how often they felt “lack companionship”, “left out”, and “isolated from others”, with response options of “never”, “sometimes”, and “always”. The total loneliness score ranged from 3 to 9, with a higher score indicating greater loneliness. Participants were considered as experiencing loneliness if they had a score ≥ 6 as previous studies did [39].

We measured depressive symptoms using the 10-item Center for Epidemiologic Studies Depression (CESD-10) Scale [40]. The CESD-10 showed good reliability and validity, with Cronbach’s α coefficients ranging from 0.73 to 0.82, and test-retest reliability coefficient of 0.71 [40–42]. The reliability and validity of the CESD-10 have also been verified in the Chinese population [43]. The 10 items evaluated the depressive behaviors and feelings of participants during the last week as follows: “Felt depressed”, “Felt everything I did was an effort”, “Sleep was restless”, “Was happy”, “Felt lonely”, “Bothered by little things”, “Could not get going”, “Had trouble keeping mind”, “Feel hopeful about the future”, and “Felt fearful”. The scores for each of the 10 items ranged from 0 to 3. The summary score of CESD-10 ranged from 0 to 30, with a higher score indicating severer depressive symptom during the last week. Participants were considered as having depressive symptom if they had a CESD-10 score ≥ 10 as previous studies did [40].

### Covariates

Data on age, sex (male, or female), marital status (currently unmarried, or married), educational level (primary school, middle and high school, or college and above), occupational status (technology, management, or others), family income per month (< 10,000 Renminbi [RMB], or ≥ 10,000 RMB), physical activity (metabolic equivalents [METs]×min/week), drinking status (current, ever, or never), smoking status (current, ever, or never), psychological disease history (yes, or no), and self-rated health status (very good, good, fair, poor, or very poor) were collected through a questionnaire interview. Physical activity was assessed by the short form of the International Physical Activity Questionnaire [44], and METs×min in the last week was calculated. Body weight and height were measured by a trained nurse through an ultrasonic device during physical examination. BMI was calculated as weight/height^2^ (kg/m^2^). We considered these covariates as they may confound the associations of perceived stress, sleep quality, with loneliness and depressive symptoms [45–52].

### Statistical analyses

The basic characteristics of the study participants were summarized in total and by poor global sleep quality. Continuous and categorical variables were presented as median (inter-quartile range [IQR]) and number (percentage), respectively. Wilcoxon rank sum tests and χ^2^ were used to compare differences by poor global sleep quality.

We first estimated the associations of perceived stress with loneliness and depressive symptoms. General linear regression models were used to estimate the associations of perceived stress score (per 1-point increment) with loneliness score and depression score (continuous variables), and adjusted β and corresponding 95% confidence intervals (CIs) were documented. Multivariable logistic regression models were used to estimate the associations of perceived stress score (per 1-point increment) with loneliness and depressive symptoms (binary variables), and adjusted odds ratios (ORs) and corresponding 95% CIs were documented. Model 1 was adjusted for age and sex. Model 2 was additionally adjusted for marital status, educational level, occupational status, family income per month, BMI, physical activity, drinking status, smoking status, psychological disease history, and self-rated health status. Model 3 was based on model 2 and further adjusted for sleep quality score. Also, the associations of perceived stress score with loneliness and depressive symptoms stratified by subgroups of poor global sleep quality were estimated. Moreover, we added a multiplication term in model 2 to calculate *P* value for interactions between perceived stress and poor global sleep quality among total participants.

Then, we performed formal mediation analyses to estimate the mediating role of sleep quality in the associations of perceived stress with loneliness and depressive symptoms. First, we estimated the associations of perceived stress with sleep quality. General linear regression models and multivariable logistic regression models were used to analyze sleep quality score and poor global sleep quality, respectively. Two models were performed: model 1 was adjusted for age and sex; and model 2 was additionally adjusted for marital status, educational level, occupational status, family income per month, BMI, physical activity, drinking status, smoking status, psychological disease history, and self-rated health status based on model 1. Second, the associations of sleep quality score with loneliness and depressive symptoms were estimated with general linear regression models and multivariable logistic regression models. The aforementioned two models were used. Similarly, setting those participants without poor global sleep quality as the reference group, we estimated the associations of poor global sleep quality with loneliness and depressive symptoms. Finally, we used the R package “*mediation*” with 1000 simulations to estimate the mediation proportions and corresponding 95% CIs after adjustment for age, sex, marital status, educational level, occupational status, family income per month, BMI, drinking status, smoking status, psychological disease history, and self-rated health status.

To test the robustness of the results and identify potentially susceptible populations, we performed subgroup analyses by age (< 45 years, ≥ 45 years), sex (male, female), educational level (below college, college and above), family income level (< 10,000 RMB per month, ≥ 10,000 RMB per month), smoking status (never, current/ever), drinking status (never, current/ever), and BMI categories (< 24 kg/m^2^, ≥ 24 kg/m^2^).

All statistical analyses were conducted using SAS version 9.4 (SAS Institute) and R version 3.6.3 (2020-02-29). A two-tailed *P* value < 0.05 was considered statistically significant.

## Results

### Basic characteristics of the participants

The median age of 734 recruited participants was 50.3 (IQR: 40.2, 58.9) years, and the majority were male (56.4%, 414/734). The median perceived stress score was 8.0 (IQR: 4.0, 12.0). About 9.9% (73/734) and 10.6% (78/734) of participants reported loneliness and depressive symptoms, respectively. The median sleep quality score was 5.0 (IQR: 3.0, 7.0), with approximately 40.6% (298/734) of participants experiencing poor global sleep quality. Adults with poor global sleep quality were more likely to be older, female, have a lower level of BMI, have poorer self-rated health status, and have a higher level of perceived stress score, sleep quality score, loneliness score, and depression score, compared with those without poor global sleep quality. Table 1 presents detailed characteristics of the total participants and by poor global sleep quality.

**Table 1:** Basic characteristics of the study participants in ZheJiang longitudinAl Study of Healthy Aging (JASHA) (*N* = 734)

Variables	Total (*N* = 734)	Poor global sleep quality		*P* value
		No (*N* = 436)	Yes (*N* = 298)	
Age in years, median (IQR)	50.3 (40.2, 58.9)	48.6 (39.7, 57.9)	52.7 (41.6, 60.0)	0.009
Sex, N (%)				< 0.001
Male	414 (56.4)	268 (61.5)	146 (49.0)	
Female	320 (43.6)	168 (38.5)	152 (51.0)	
Educational level, N (%)				0.794
Primary school	67 (9.1)	38 (8.7)	29 (9.7)	
Middle and high school	327 (44.6)	192 (44.0)	135 (45.3)	
College and above	340 (46.3)	206 (47.2)	134 (45.0)	
Marital status, N (%)				0.123
Currently unmarried	69 (9.4)	35 (8.0)	34 (11.4)	
Currently married	665 (90.6)	401 (92.0)	264 (88.6)	
Occupational status, N (%)				0.642
Technology	126 (17.2)	72 (16.5)	54 (18.1)	
Management	134 (18.3)	84 (19.3)	50 (16.8)	
Others	474 (64.6)	280 (64.2)	194 (65.1)	
Family income per month, N (%)				0.150
< 10,000 RMB	251 (34.2)	140 (32.1)	111 (37.2)	
≥ 10,000 RMB	483 (65.8)	296 (67.9)	187 (62.8)	
Smoking status, N (%)				0.590
Never	527 (71.8)	307 (70.4)	220 (73.8)	
Current	147 (20.0)	91 (20.9)	56 (18.8)	
Ever	60 (8.2)	38 (8.7)	22 (7.4)	
Drinking status, N (%)				0.641
Current	286 (39.0)	173 (39.7)	113 (37.9)	
Ever	20 (2.7)	10 (2.3)	10 (3.4)	
Never	428 (58.3)	253 (58.0)	175 (58.7)	
BMI in kg/m^2^, median (IQR)	23.9 (21.5, 26.3)	24.2 (21.8, 26.5)	23.2 (21.1, 26.1)	0.004
Physical activity (METs×min/week), median (IQR)	2079.0 (1080.0, 3732.0)	2079.0 (1114.5, 3824.4)	2085.5 (1044.0, 3657.0)	0.604
Psychological disease history, N (%)				0.160
No	730 (99.5)	435 (99.8)	295 (99.0)	
Yes	4 (0.5)	1 (0.2)	3 (1.0)	
Self-rated health status, N (%)				< 0.001
Very good	26 (3.5)	21 (4.8)	5 (1.7)	
Good	273 (37.2)	180 (41.3)	93 (31.2)	
Fair	360 (49.0)	202 (46.3)	158 (53.0)	
Poor	73 (9.9)	33 (7.6)	40 (13.4)	
Very poor	2 (0.3)	0 (0.0)	2 (0.7)	
Perceived stress score, median (IQR)	8.0 (4.0, 12.0)	7.0 (4.0, 11.0)	9.0 (5.0, 13.0)	< 0.001
Sleep quality score, median (IQR)	5.0 (3.0, 7.0)	3.0 (2.0, 4.0)	8.0 (6.0, 10.0)	< 0.001
Subjective sleep quality score, median (IQR)	1.0 (1.0, 2.0)	1.0 (1.0, 1.0)	2.0 (1.0, 2.0)	< 0.001
Sleep latency score, median (IQR)	1.0 (0.0, 1.0)	0.0 (0.0, 1.0)	1.0 (1.0, 2.0)	< 0.001
Sleep duration score, median (IQR)	1.0 (0.0, 2.0)	0.0 (0.0, 1.0)	2.0 (1.0, 3.0)	< 0.001
Sleep efficiency score, median (IQR)	0.0 (0.0, 1.0)	0.0 (0.0, 0.0)	1.0 (0.0, 2.0)	< 0.001
Sleep disturbances score, median (IQR)	1.0 (0.0, 1.0)	0.0 (0.0, 1.0)	1.0 (1.0, 1.0)	< 0.001
Use of sleep medicine score, median (IQR)	0.0 (0.0, 0.0)	0.0 (0.0, 0.0)	0.0 (0.0, 0.0)	< 0.001
Daytime dysfunction score, median (IQR)	1.0 (0.0, 2.0)	0.0 (0.0, 1.0)	2.0 (1.0, 3.0)	< 0.001
Loneliness score, median (IQR)	3.0 (3.0, 4.0)	3.0 (3.0, 4.0)	3.0 (3.0, 5.0)	< 0.001
Loneliness, N (%)				0.019
No (Loneliness score < 6)	661 (90.1)	402 (92.2)	259 (86.9)	
Yes (Loneliness score ≥ 6)	73 (9.9)	34 (7.8)	39 (13.1)	
Depression score, median (IQR)	4.0 (2.0, 7.0)	3.0 (1.0, 5.0)	5.0 (3.0, 8.0)	< 0.001
Depressive symptoms, N (%)				< 0.001
No (Depression score < 10)	656 (89.4)	415 (95.2)	241 (80.9)	
Yes (Depression score ≥ 10)	78 (10.6)	21 (4.8)	57 (19.1)	

IQR, inter-quartile range; RMB, Renminbi; BMI, body mass index; METs, metabolic equivalents

### Associations of perceived stress with loneliness and depressive symptoms

Table 2 reports the associations of perceived stress with loneliness and depressive symptoms. We found that with each 1-point increment in the perceived stress score, both the loneliness score (β = 0.07; 95% CI: 0.06, 0.08) and depression score (β = 0.45; 95% CI: 0.40, 0.49) increased significantly in the age- and sex-adjusted model. After further adjusting for marital status, educational level, occupational status, family income per month, BMI, physical activity, drinking status, smoking status, psychological disease history, self-rated health status, and sleep quality score (model 2, and model 3), these positive associations remained. Additionally, we observed similar results for the associations of perceived stress score with loneliness and depressive symptoms. For instance, with each 1-point increment in perceived stress score, the multivariable-adjusted ORs for loneliness and depressive symptoms were 1.16 (95% CI: 1.10, 1.22), and 1.35 (95% CI: 1.26, 1.44), respectively, in the fully adjusted model.

**Table 2:** Associations of perceived stress score (per 1-point increment) with loneliness and depressive symptoms and the mediation proportion of perceived stress in loneliness and depressive symptoms attributed to sleep quality (*N* = 734)

Outcomes	β/OR (95% CI)			Mediation proportion, % (95% CI) ^d^	*P* value
	Model 1 ^a^	Model 2 ^b^	Model 3 ^c^		
Loneliness score	0.07 (0.06, 0.08)	0.07 (0.05, 0.08)	0.06 (0.05, 0.08)	5.3 (1.3, 10.0)	0.014
Depression score	0.45 (0.40, 0.49)	0.42 (0.38, 0.46)	0.38 (0.34, 0.42)	9.7 (6.2, 14.0)	< 0.001
Loneliness ^e^	1.17 (1.11, 1.22)	1.17 (1.11, 1.23)	1.16 (1.10, 1.22)	4.3 (-1.9, 13.0)	0.170
Depressive symptoms ^f^	1.34 (1.26, 1.42)	1.36 (1.27, 1.45)	1.35 (1.26, 1.44)	5.8 (2.2, 11.0)	< 0.001

OR, odds ratio; CI, confidence interval

^a^ Model 1 was adjusted for age, and sex

^b^ Model 2 was further adjusted for marital status, educational level, occupational status, family income per month, body mass index, physical activity, drinking status, smoking status, psychological disease history, and self-rated health status based on model 1

^c^ Model 3 was further adjusted for the Pittsburgh Sleep Quality Index score based on model 2

^d^ The model was adjusted for age, sex, marital status, educational level, occupational status, family income per month, body mass index, physical activity, drinking status, smoking status, psychological disease history, and self-rated health status

^e^ Participants were considered as experiencing loneliness if they had a loneliness score ≥ 6

^f^ Participants were considered as having depressive symptoms if they had a depression score ≥ 10

The associations of perceived stress with loneliness and depressive symptoms stratified by subgroups of poor global sleep quality were estimated (Table 3), and no remarkable changes in results were observed. We found interactions of perceived stress with poor global sleep quality (*P* = 0.002), with a stronger magnitude of the associations between perceived stress and depression score among participants with poor global sleep quality (β = 0.45; 95% CI: 0.38, 0.52) than among those who did not experience poor global sleep quality (β = 0.33; 95% CI: 0.28, 0.39).

**Table 3:** Associations of perceived stress score (per 1-point increment) with loneliness and depressive symptoms stratified by poor global sleep quality (*N* = 734). ^a^

Outcomes	Poor global sleep quality	β/OR (95% CI)	*P* value for interaction ^b^
Loneliness score	No (*N* = 436)	0.07 (0.05, 0.09)	0.721
	Ye (*N* = 298)	0.06 (0.03, 0.08)	
Depression score	No (*N* = 436)	0.33 (0.28, 0.39)	0.002
	Yes (*N* = 298)	0.45 (0.38, 0.52)	
Loneliness ^c^	No (*N* = 436)	1.23 (1.14, 1.33)	0.156
	Yes (*N* = 298)	1.11 (1.03, 1.19)	
Depressive symptoms ^d^	No (*N* = 436)	1.37 (1.20, 1.55)	0.344
	Yes (*N* = 298)	1.40 (1.26, 1.55)	

OR, odds ratio; CI, confidence interval

^a^ The models were adjusted for age, sex, marital status, educational level, occupational status, family income per month, body mass index, physical activity, drinking status, smoking status, psychological disease history, and self-rated health status

^b^*P* value for interaction indicated the modifying effect of poor global sleep quality on the associations of perceived stress with loneliness and depressive symptoms among total participants

^c^ Participants were considered as experiencing loneliness if they had a loneliness score ≥ 6

^d^ Participants were considered as having depressive symptoms if they had a depression score ≥ 10

### Mediation analyses of sleep quality in the associations of perceived stress with loneliness and depressive symptoms

First, we observed the significant associations of perceived stress with sleep quality (Additional file 1: Table S1). With adjustment for age and sex (model 1), the perceived stress score was positively associated with the sleep quality score (β = 0.15; 95% CI: 0.11, 0.20). After adjusting for more confounding factors (model 2), the significant results remained. Moreover, each 1-point increment in perceived stress score increased the odds of poor global sleep quality by 7% (OR = 1.07; 95% CI: 1.04, 1.10) (model 2).

Second, the positive associations of sleep quality with loneliness and depressive symptoms were also observed (Additional file 1: Table S2-S3). For instance, with each 1-point increment in sleep quality score, the odds of loneliness and depressive symptoms increased by 10% (OR = 1.10; 95% CI: 1.04, 1.19) and 21% (OR = 1.21; 95% CI: 1.13, 1.30) (Additional file 1: Table S2), respectively. Compared with those without poor global sleep quality, participants with poor global sleep quality exhibited higher odds of loneliness and depressive symptoms, with ORs of 1.82 (95% CI: 1.09, 3.04) and 4.53 (95% CI: 2.55, 8.04), respectively (Additional file 1: Table S3).

Finally, the results of formal mediation analyses are shown in Table 2. We observed that sleep quality significantly mediated 5.3% (95% CI: 1.3%, 10.0%; *P* = 0.014) and 9.7% (95% CI: 6.2%, 14.0%; *P* < 0.001) of the associations of perceived stress score with loneliness score and depression score, respectively. Furthermore, sleep quality mediated 5.8% (95% CI: 2.2, 11.0; *P* < 0.001) of the effect of perceived stress on depressive symptoms. Nevertheless, we did not find a significant mediation role of sleep quality in the association between perceived stress and loneliness (*P* = 0.170).

Additionally, in the subgroup analyses, we found that the associations of perceived stress with loneliness and depressive symptoms remained significant (Additional file 1: Table S4). In most of subgroups, sleep quality partially mediated these associations.

## Discussion

Based on a sample of general adults aged over 18 years from China, we demonstrated that perceived stress and poor sleep quality were positively associated with loneliness and depressive symptoms. Furthermore, sleep quality partially mediated the associations of perceived stress with loneliness and depressive symptoms. The findings reveal a pathway linking perceived stress to loneliness and depressive symptoms, and highlight the importance of reducing stress and improving sleep quality in promoting mental health.

It has been reported that there were differences in emotional regulation related to mental health due to cultural context [53]. Previous studies have suggested a positive association between perceived stress and depressive symptoms across different countries [21, 22]. However, only a few studies focus on global stress levels [22] rather than stress induced by specific negative life events (e.g., financial problems, serious illness) [20, 54]. The global perception of stress emphasizes the subjective assessment of stress and coping ability, which is consistent with the original concept of stress [55]. Measuring the extent to which individuals self-evaluate their life experiences as stressful helps to fully understand the associations between stress and depressive symptoms. The present study extended such explorations using PSS-10 among the general adults in China and observed consistent results. Furthermore, we linked global perceived stress to loneliness in this study and observed similar results. Loneliness has become a major concern globally, particularly after the COVID-19 pandemic [9]. In a recent systematic review and meta-analysis, substantial geographical variation in loneliness prevalence has been reported [56]. China is a developing country with vast land and rich resources, exploring the association between perceived stress and loneliness in Chinese adults is needed. In line with previous studies [17, 18], our results show that a higher level of perceived stress is associated with higher odds of loneliness. Loneliness may be induced by exaggerated responses to acute stress, particularly for blood pressure and inflammation [57]. It is worth noting that Chinese cultural beliefs and Chinese beliefs about traditional medicine affect understanding of depression, illness management, and social interaction among adults with depression [24]. For instance, Chinese adults with mental health problems are likely to hide personal emotion and avoid self-reporting of stress and depressive symptoms. Our findings highlight the importance of reducing stress in eliminating loneliness and depressive symptoms among Chinese adults, although these associations need to be confirmed in prospective studies and randomized control trials.

The findings that poor sleep quality partially mediated the associations of perceived stress with loneliness and depressive symptoms in general Chinese adults provide clues for behavioral mechanisms linking stress to mental health. High levels of psychological stress may induce emotional reactivity, physiological activation, and arousal before bedtime, thereby leading to deteriorating sleep quality [25, 58, 59]. Several studies have demonstrated that adults who perceive higher levels of stress are more likely to report poor sleep quality [25, 26]. In addition, sleep behaviors are found to predict loneliness [60] and depressive symptoms [28, 61] in epidemiological studies. Poor sleep quality may cause changes in emotional reactivity [62], which in turn interacts with the regulation of homeostatic and circadian rhythms [63], thus affecting mental health [64]. The evidence supports our hypothesis that perceived stress is associated with loneliness and depressive symptoms through impaired sleep behaviors. Our findings extend previous studies in specific populations (i.e., urban older adults, students, or health workers) [29–32] to the general adults and focus on both loneliness and depressive symptoms. The findings imply us that sleep intervention programs may help promoting mental health among adults. More importantly, non-pharmacological interventions, such as exercise [46] and acupressure [65], have been shown to improve sleep quality in randomized controlled trials, indicating the feasibility and effectiveness of intervention strategies targeting sleep. Existing evidence also supports the efficacy of these non-pharmacological interventions in Chinese adults [66–68]. Our findings prompt policy makers to prioritize tailored and feasible sleep interventions for highly stressed adults to improve sleep quality, and further promote mental health. Particularly, in the context of post-pandemic mental health challenges, it is clear that the implementation of targeted intervention strategies is critical for the promotion of mental well-being.

The strengths of this study include the sample of general adults aged over 18 years from China, and the assessment of global perceived stress. Nevertheless, there are also several limitations. First, because of the cross-sectional study design, the results should be interpreted cautiously and we cannot draw causal conclusions. Future prospective cohort studies and interventional trials are required to validate our results. Second, perceived stress, sleep quality, as well as loneliness, and depressive symptoms were all self-reported and only measured once, while they may change over time. Further studies are warranted to assess their complex associations through repeated measurements. Third, China is a country with vast land and a large population. However, our study only included a sample from a single city in China, so that the observed associations may lack generalizability to some extent. Fourth, although we controlled for many covariates, residual confounding is still unavoidable.

## Conclusion

In a sample of general Chinese adults, a higher level of perceived stress was associated with higher odds of loneliness and depressive symptoms, and sleep quality partially mediated these associations. The findings reveal a potential pathway from perceived stress to mental health through sleep behaviors, and underscore the potential of sleep intervention programs to promote mental health among adults who feel highly stressed.

### Electronic supplementary material

Below is the link to the electronic supplementary material.

Supplementary Material 1

## Data Availability

The datasets used and/or analysed during the current study are available from the corresponding author on reasonable request.

## Abbreviations

BMI: Body mass index
CESD-10: 10-item Center for Epidemiologic Studies Depression
CI: Confidence interval
COVID-19: Coronavirus disease 2019
IQR: Inter-quartile range
JASHA: The ZheJiang longitudinAl Study of Healthy Aging
METs: Metabolic equivalents
OR: Odds ratio
PSQI: Pittsburgh Sleep Quality Index
PSS-10: 10-item Perceived Stress Scale
RMB: Renminbi
UCLA: University of California, Los Angeles
WHO: World Health Organization
