# Supplementary material for: Associations of perceived stress with loneliness and depressive symptoms: the mediating role of sleep quality
Source: BMC Psychiatry. 2024 Mar 1;24:172. doi: 10.1186/s12888-024-05609-2 (PMC10905934; doi:10.1186/s12888-024-05609-2)
Supplement: Supplementary file 1 — Supplementary Material 1 [file 12888_2024_5609_MOESM1_ESM.docx]

**Additional file 1**

**Table S1. Associations of perceived stress score (per 1-point increment) with sleep quality (N=734).**

| **Outcomes** | **Model 1 ^a^** | **Model 2 ^b^** |
| --- | --- | --- |
|  | **β (95% CI)** | |
| **Sleep quality score** | 0.15 (0.11, 0.20) | 0.13 (0.08, 0.18) |
|  | **OR (95% CI)** | |
| **Poor global sleep quality ^c^** | 1.08 (1.05, 1.11) | 1.07 (1.04, 1.10) |

CI, confidence interval; OR, odds ratio.

^a^ Model 1 was adjusted for age, and sex.
^b^ Model 2 was further adjusted for marital status, educational level, occupational status, family income per month, body mass index, physical activity, drinking status, smoking status, psychological disease history, and self-rated health status based on model 1.

^c^ Participants were considered as having poor global sleep quality if they had a sleep quality score >5.

**Table S2. Associations of sleep quality score (per 1-point increment) with loneliness and depressive symptoms (N=734).**

| **Outcomes** | **Model 1 ^a^** | **Model 2 ^b^** |
| --- | --- | --- |
|  | **β (95% CI)** | |
| **Loneliness score** | 0.06 (0.03, 0.08) | 0.05 (0.03, 0.07) |
| **Depression score** | 0.50 (0.42, 0.57) | 0.43 (0.36, 0.51) |
|  | **OR (95% CI)** | |
| **Loneliness ^c^** | 1.12 (1.05, 1.20) | 1.10 (1.04, 1.19) |
| **Depressive symptoms ^d^** | 1.25 (1.17, 1.33) | 1.21 (1.13, 1.30) |

CI, confidence interval; OR, odds ratio.

^a^ Model 1 was adjusted for age, and sex.
^b^ Model 2 was further adjusted for marital status, educational level, occupational status, family income per month, body mass index, physical activity, drinking status, smoking status, psychological disease history, and self-rated health status based on model 1.

^c^ Participants were considered as experiencing loneliness if they had a loneliness score ≥6.

^d^ Participants were considered as having depressive symptoms if they had a depression score ≥10.

**Table S3. Associations of poor global sleep quality with loneliness and depressive symptoms (N=734).**

| **Outcomes** | **Model 1 ^a^** | **Model 2 ^b^** |
| --- | --- | --- |
|  | **β (95% CI)** | |
| **Loneliness score** | 0.32 (0.17, 0.47) | 0.28 (0.13, 0.44) |
| **Depression score** | 3.09 (2.56, 3.61) | 2.72 (2.20, 3.24) |
|  | **OR (95% CI)** | |
| **Loneliness ^c^** | 1.95 (1.19, 3.20) | 1.82 (1.09, 3.04) |
| **Depressive symptoms ^d^** | 5.28 (3.08, 9.04) | 4.53 (2.55, 8.04) |

CI, confidence interval; CESD-10, 10-item Center for Epidemiologic Studies Depression Scale; OR, odds ratio.

^a^ Model 1 was adjusted for age, and sex.
^b^ Model 2 was further adjusted for marital status, educational level, occupational status, family income per month, body mass index, physical activity, drinking status, smoking status, psychological disease history, and self-rated health status based on model 1.

^c^ Participants were considered as experiencing loneliness if they had a loneliness score ≥6.

^d^ Participants were considered as having depressive symptoms if they had a depression score ≥10.

**Table S4. Associations of perceived stress score (per 1-point increment) with loneliness and depressive symptoms and the mediation proportion of perceived stress in loneliness and depressive symptoms attributed to sleep quality in population subgroups.**

| **Subgroups** | | **Outcomes** | **β/OR (95% CI)** | | **P value for interaction ^c^** | **Mediation proportion,**  **% (95% CI) ^d^** | **P value** |
| --- | --- | --- | --- | --- | --- | --- | --- |
|  |  |  | **Model 1 ^a^** | **Model 2 ^b^** |  |  |  |
| **By age** | **<45 years** | **Loneliness score** | 0.07 (0.05, 0.10) | 0.06 (0.03, 0.09) | 0.820 | 17.4 (5.6, 36.0) | 0.004 |
|  | **≥45 years** |  | 0.07 (0.05, 0.08) | 0.07 (0.05, 0.08) |  | 1.4 (-1.3, 6.0) | 0.270 |
|  | **<45 years** | **Depression score** | 0.51 (0.43, 0.58) | 0.45 (0.37, 0.52) | 0.035 | 11.8 (5.9, 19.0) | <0.001 |
|  | **≥45 years** |  | 0.37 (0.32, 0.42) | 0.34 (0.29, 0.39) |  | 6.5 (1.3, 12.0) | 0.024 |
|  | **<45 years** | **Loneliness ^e^** | 1.20 (1.10, 1.31) | 1.18 (1.08, 1.29) | 0.786 | 8.1 (-10.2, 30.0) | 0.340 |
|  | **≥45 years** |  | 1.17 (1.10, 1.25) | 1.17 (1.09, 1.24) |  | 1.8 (-3.2, 10.0) | 0.450 |
|  | **<45 years** | **Depressive symptoms ^f^** | 1.36 (1.22, 1.51) | 1.34 (1.19, 1.50) | 0.526 | 5.4 (-5.3, 18.0) | 0.310 |
|  | **≥45 years** |  | 1.39 (1.27, 1.53) | 1.40 (1.27, 1.56) |  | 4.6 (0.9, 10.0) | 0.016 |
| **By sex** | **Male** | **Loneliness score** | 0.06 (0.04, 0.08) | 0.06 (0.04, 0.08) | 0.276 | 3.6 (-2.2, 12.0) | 0.250 |
|  | **Female** |  | 0.07 (0.05,0.09) | 0.07 (0.05, 0.09) |  | 8.0 (1.8, 18.0) | 0.010 |
|  | **Male** | **Depression score** | 0.40 (0.34, 0.45) | 0.35 (0.30, 0.40) | 0.232 | 12.0 (6.9, 18.0) | <0.001 |
|  | **Female** |  | 0.45 (0.39, 0.52) | 0.41 (0.35, 0.47) |  | 9.1 (3.8, 15.0) | <0.001 |
|  | **Male** | **Loneliness ^e^** | 1.15 (1.08, 1.22) | 1.13 (1.06, 1.21) | 0.649 | 6.3 (-4.8, 23.0) | 0.220 |
|  | **Female** |  | 1.20 (1.11, 1.30) | 1.19 (1.10, 1.29) |  | 4.7 (-3.5, 17.0) | 0.290 |
|  | **Male** | **Depressive symptoms ^f^** | 1.34 (1.22, 1.47) | 1.32 (1.19, 1.46) | 0.426 | 8.7 (2.6, 17.0) | 0.006 |
|  | **Female** |  | 1.39 (1.25, 1.54) | 1.37 (1.23, 1.52) |  | 4.8 (-0.2, 13.0) | 0.064 |
| **By educational level** | **Below college** | **Loneliness score** | 0.07 (0.06, 0.09) | 0.07 (0.05, 0.09) | 0.383 | 5.4 (-1.0, 13.0) | 0.100 |
|  | **College and above** |  | 0.06 (0.04, 0.09) | 0.06 (0.04, 0.08) |  | 4.3 (-0.3, 10.0) | 0.074 |
|  | **Below college** | **Depression score** | 0.40 (0.34, 0.46) | 0.36 (0.30, 0.41) | 0.362 | 10.4 (5.7, 16.0) | <0.001 |
|  | **College and above** |  | 0.46 (0.40, 0.53) | 0.43 (0.36, 0.49) |  | 8.2 (3.2, 14.0) | <0.001 |
|  | **Below college** | **Loneliness ^e^** | 1.18 (1.10, 1.27) | 1.18 (1.10, 1.27) | 0.504 | 1.4 (-8.0, 12.0) | 0.750 |
|  | **College and above** |  | 1.15 (1.07, 1.24) | 1.14 (1.06, 1.23) |  | 4.2 (-7.5, 20.0) | 0.420 |
|  | **Below college** | **Depressive symptoms ^f^** | 1.37 (1.25, 1.50) | 1.36 (1.23, 1.49) | 0.658 | 6.9 (1.4, 14.0) | 0.010 |
|  | **College and above** |  | 1.43 (1.27, 1.60) | 1.40 (1.24, 1.57) |  | 3.3 (-1.3, 10.0) | 0.160 |
| **By family income level** | <10000 RMB | **Loneliness score** | 0.06 (0.04, 0.09) | 0.06 (0.04, 0.08) | 0.805 | 4.8 (-1.1, 16.0) | 0.110 |
|  | ≥10000 RMB |  | 0.07 (0.05, 0.09) | 0.06 (0.05, 0.08) |  | 6.7 (0.7, 16.0) | 0.032 |
|  | <10000 RMB | **Depression score** | 0.42 (0.35, 0.49) | 0.39 (0.32, 0.45) | 0.823 | 7.7 (1.8, 15.0) | 0.008 |
|  | ≥10000 RMB |  | 0.42 (0.37, 0.48) | 0.37 (0.32, 0.43) |  | 11.8 (7.0, 17.0) | <0.001 |
|  | <10000 RMB | **Loneliness ^e^** | 1.19 (1.09, 1.31) | 1.19 (1.08, 1.31) | 0.671 | 8.7 (2.0, 25.0) | 0.042 |
|  | ≥10000 RMB |  | 1.17 (1.10, 1.25) | 1.17 (1.10, 1.25) |  | 0.2 (-11.8, 12.0) | 0.999 |
|  | <10000 RMB | **Depressive symptoms ^f^** | 1.44 (1.26, 1.64) | 1.45 (1.26, 1.66) | 0.912 | 4.7 (1.8, 13.0) | 0.048 |
|  | ≥10000 RMB |  | 1.37 (1.25, 1.51) | 1.35 (1.22, 1.49) |  | 8.4 (2.9, 16.0) | 0.004 |
| **By drinking status** | Never | **Loneliness score** | 0.07 (0.06, 0.09) | 0.07 (0.05, 0.09) | 0.411 | 5.8 (1.0, 13.0) | 0.018 |
|  | Current/Ever |  | 0.06 (0.04, 0.08) | 0.06 (0.03, 0.08) |  | 2.4 (-5.5, 12.0) | 0.530 |
|  | Never | **Depression score** | 0.47 (0.42, 0.53) | 0.43 (0.38, 0.48) | 0.006 | 8.6 (4.6, 14.0) | <0.001 |
|  | Current/Ever |  | 0.34 (0.27, 0.40) | 0.29 (0.23, 0.35) |  | 13.6 (6.6, 22.0) | <0.001 |
|  | Never | **Loneliness ^e^** | 1.16 (1.09, 1.23) | 1.15 (1.08, 1.23) | 0.163 | 4.0 (-4.0, 14.0) | 0.330 |
|  | Current/Ever |  | 1.22 (1.10, 1.35) | 1.21 (1.09, 1.34) |  | 5.2 (-5.0, 22.0) | 0.250 |
|  | Never | **Depressive symptoms ^f^** | 1.39 (1.28, 1.52) | 1.38 (1.26, 1.51) | 0.160 | 5.4 (1.4, 12.0) | 0.012 |
|  | Current/Ever |  | 1.29 (1.15, 1.45) | 1.26 (1.11, 1.43) |  | 11.1 (2.1, 28.0) | 0.008 |
| **By smoking stats** | Never | **Loneliness score** | 0.07 (0.06, 0.09) | 0.07 (0.05, 0.09) | 0.115 | 5.3 (0.8, 12.0) | 0.006 |
|  | Current/Ever |  | 0.06 (0.03, 0.08) | 0.05 (0.03, 0.08) |  | 7.0 (-3.8, 23.0) | 0.188 |
|  | Never | **Depression score** | 0.43 (0.38, 0.48) | 0.39 (0.34, 0.43) | 0.854 | 9.6 (5.7, 15.0) | <0.001 |
|  | Current/Ever |  | 0.42 (0.34, 0.50) | 0.37 (0.29, 0.45) |  | 11.1 (4.4, 20.0) | 0.004 |
|  | Never | **Loneliness ^e^** | 1.19 (1.12, 1.26) | 1.18 (1.11, 1.25) | 0.531 | 2.5 (-4.1, 11.0) | 0.450 |
|  | Current/Ever |  | 1.15 (1.05, 1.27) | 1.12 (1.01, 1.24) |  | 17.9 (-1.1, 70.0) | 0.072 |
|  | Never | **Depressive symptoms ^f^** | 1.35 (1.23, 1.45) | 1.34 (1.23, 1.45) | 0.606 | 6.1 (1.8, 13.0) | 0.010 |
|  | Current/Ever |  | 1.55 (1.28, 1.89) | 1.61 (1.27, 2.03) |  | 7.1 (0.7, 17.0) | 0.034 |
| **By BMI categories** | <24kg/m^2^ | **Loneliness score** | 0.07 (0.05, 0.09) | 0.07 (0.05, 0.09) | 0.786 | 5.3 (-0.8, 14.0) | 0.082 |
|  | ≥24kg/m^2^ |  | 0.07 (0.05, 0.09) | 0.06 (0.05, 0.08) |  | 4.5 (-0.1, 12.0) | 0.058 |
|  | <24kg/m^2^ | **Depression score** | 0.44 (0.37, 0.50) | 0.39 (0.33, 0.45) | 0.964 | 11.0 (5.6, 17.0) | <0.001 |
|  | ≥24kg/m^2^ |  | 0.41 (0.35, 0.47) | 0.38 (0.32, 0.43) |  | 7.5 (2.6, 13.0) | 0.010 |
|  | <24kg/m^2^ | **Loneliness ^e^** | 1.16 (1.08, 1.24) | 1.16 (1.08, 1.24) | 0.144 | 1.7 (-9.2, 16.0) | 0.750 |
|  | ≥24kg/m^2^ |  | 1.22 (1.13, 1.32) | 1.21 (1.11, 1.31) |  | 5.3 (0.07, 15.0) | 0.048 |
|  | <24kg/m^2^ | **Depressive symptoms ^f^** | 1.42 (1.28, 1.58) | 1.40 (1.26, 1.56) | 0.880 | 5.8 (0.4, 13.0) | 0.040 |
|  | ≥24kg/m^2^ |  | 1.38 (1.23, 1.54) | 1.38 (1.23, 1.54) |  | 3.5 (-0.2, 10.0) | 0.080 |

OR, odds ratio; CI, confidence interval; RMB, Renminbi; BMI, body mass index.
^a^ Model 1 was adjusted for age, sex (not included in the subgroup analyses by sex), marital status, educational level (not included in the subgroup analyses by educational level), occupational status, family income per month (not included in the subgroup analyses by family income level), body mass index, physical activity, drinking status (not included in the subgroup analyses by drinking status), smoking status (not included in the subgroup analyses by smoking status), psychological disease history, and self-rated health status.
^b^ Model 2 was further adjusted for the Pittsburgh Sleep Quality Index score based on model 1.
^c^ The model was adjusted for age, sex, marital status, educational level, occupational status, family income per month, body mass index, physical activity, drinking status, smoking status, psychological disease history, and self-rated health status. P value for interaction indicated the modifying effect of subgroups on the associations of perceived stress with loneliness and depressive symptoms among total participants.

^d^ The model was adjusted for age, sex (not included in the subgroup analyses by sex), marital status, educational level (not included in the subgroup analyses by educational level), occupational status, family income per month (not included in the subgroup analyses by family income level), body mass index, physical activity, drinking status (not included in the subgroup analyses by drinking status), smoking status (not included in the subgroup analyses by smoking status), psychological disease history, and self-rated health status.

^e^ Participants were considered as experiencing loneliness if they had a loneliness score ≥6.

^f^ Participants were considered as having depressive symptoms if they had a depression score ≥10.
